# Editorial: Linking cellular metabolism to hematological malignancies

**DOI:** 10.3389/fonc.2022.1077358

**Published:** 2022-11-11

**Authors:** Xiujing He, Manoj Kumar Kashyap, Zhizhuang Joe Zhao, Jian Yu, Hubing Shi

**Affiliations:** ^1^ Laboratory of Integrative Medicine, Clinical Research Center for Breast, State Key Laboratory of Biotherapy, West China Hospital, Sichuan University and Collaborative Innovation Center, Chengdu, Sichuan, China; ^2^ Amity Stem Cell Institute, Amity Medical School, Amity University Haryana, Panchgaon (Manesar), Gurugram, Haryana-122413, India; ^3^ Department of Pathology, University of Oklahoma Health Sciences Center, Oklahoma, OK, United States; ^4^ Interdisciplinary Institute of Cancer Diagnosis and Treatment, Beijing Advanced Innovation Center for Biomedical Engineering, Beihang University, Beijing, China

**Keywords:** hematological malignancies, cellular metabolism, metabolic reprogramming, immunometabolism, drug resistance

Convincing evidence has revealed that metabolic reprogramming orchestrates tumor initiation and progression, immune evasion, and drug resistance. Targeting metabolic vulnerabilities of tumors has remarkable advantages as a therapeutic strategy that exerts prominent antitumor effects, without affecting normal cell physiology. Indeed, the current treatment options based on metabolic reprogramming present impressive curative effects in solid tumors and hematological malignancies. A better understanding of tumor metabolic remodeling and immunometabolism will deepen the insights into disease mechanisms and promote the development of promising therapeutic strategies. This Research Topic intends to highlight the current understanding of the role of cellular metabolism in hematological malignancies, with the purpose of identifying new therapeutic targets and rational metabolic therapies alone or in combination with other regimens.

## The mechanism underlying metabolic reprogramming

Arginine plays a multifaceted role in numerous crucial biological processes and exerts a significant impact on carcinogenesis and immune response. Arginine auxotrophic tumors, including many hematological malignancies, lose the ability to endogenously synthesize arginine; thus, arginine depletion therapy can serve as a promising antitumor therapeutic approach. Arginase is a prevalent arginine-depleting agent in clinical practice that converts arginine into ornithine. Du et al. illustrate the arginine metabolism pathway and summarize the current state of arginase application in the treatment of hematological malignancies. The combination of acupuncture and bortezomib is a promising therapeutic strategy to benefit multiple myeloma (MM) model mice, as outlined by Ke et al. The synergistic effects of acupuncture and bortezomib on the survival of MM mice might depend on ornithine-mediated metabolism, which is implicated in arginine and proline metabolic pathways. Furthermore, the external data from the Gene Expression Omnibus (GEO) database revealed that higher ornithine decarboxylase 1 (ODC1) expression was significantly associated with inferior prognosis in MM patients.

Emerging metabolic pathways provide new clues for identifying potential drivers of pathogenesis in hematological malignancies. As a novel characterized modality of regulatory cell death, ferroptosis has been demonstrated to be related to multiple metabolic pathways. Lan et al. summarize the recent progress in the regulatory mechanism of ferroptosis and elucidate the roles of ferroptosis in hematological malignancies. The potential of ferroptosis as a therapeutic target in hematological malignancies is further discussed.

Recent studies have revealed metabolic differences between hematopoietic and leukemic stem cells. Patel et al. depict the unique metabolic adaptations seen in leukemic stem cells (LSCs). They also highlight the advances and limitations of metabolic analysis to decipher the metabolic adaptability of LSCs. Cell number limitation and metabolic heterogeneity represent the major challenges to understanding metabolic differences in the cells of interest. Single-cell metabolic technologies provide an excellent opportunity to dissect cellular states at unprecedented depth. Zuo et al. introduce single-cell metabolomics techniques, providing clear navigation to select appropriate approaches for metabolic analysis.

## Immunometabolism in the tumor microenvironment

To date, increasing evidence indicates that immune cells can alter metabolic programs to sustain their unique phenotype and function in order to cope with environmental changes. Liu et al. explore the metabolic abnormalities of tumor-associated macrophages in CD5-positive non-MYC/BCL2 double expressor lymphoma (CD5^+^ non-DE DLBCL). They found enhanced lipid metabolism in CD5^+^ non-DE DLBCL, accompanied by an increased M2 proportion. Targeting dysregulated lipid metabolism with metformin was able to significantly decrease M2 proportion and dampen fatty acid transporter (CD36) expression *in vitro*, indicating the clinical rationale of metformin for therapeutic treatment in CD5^+^ non-DE DLBCL. Metabolic reprogramming existing in the tumor microenvironment (TME) can also influence the function of immune cells. Ehlers et al. found that activated natural killer (NK) cells can maintain cytotoxic capacity against tumor cells in a reduced glucose environment *in vitro*, as found in bone marrow of multiple myeloma (MM) patients, suggesting the therapeutic potential of activated NK cells for the treatment of MM.

## Emerging therapeutic strategies and drug resistance

Increasing efforts are being made to develop novel antitumor strategies for hematological malignancies. Lv et al. focus on treatment advances in lymphoproliferative diseases driven by Epstein Barr virus (EBV-LPDs). The traditional treatment approaches for EBV-LPDs are summarized in this review, including chemotherapy, radiotherapy, and hematopoietic stem cell transplantation. They then highlight research advances regarding alternative treatment modalities based on pathological mechanisms, such as immunotherapy, gene therapy, and epigenetic therapy. In addition, proteasome inhibitors, selective nuclear export protein inhibitors, and JAK inhibitors have been proposed as potential therapeutic options for EBV-LPDs. Zhang et al. briefly summarize the current standard treatment for classical Hodgkin lymphoma (cHL). Meanwhile, they survey the latest clinical trials of novel therapeutic agents with the potential to overcome relapsed or refractory cHL, providing promising directions for future research in this area. Glucose metabolism not only affects tumor cell growth but also contributes to shaping the immunosuppressive microenvironment, ultimately promoting immune evasion. Targeting glucose metabolism to remodel the TME is a rational strategy to enhance the efficacy of cancer immunotherapy. Liu et al. review the advances in targeting glucose metabolism combined with cancer immunotherapy.

Drug resistance remains a major obstacle in the clinical management of solid tumors and hematological malignancies. Elucidating the mechanisms of drug resistance is essential to tackle this issue. To explore the potential mechanism of resistance to arsenic trioxide (ATO) in chronic myeloid leukemia (CML), Wang et al. utilized gene chip to profile the metabolic characteristics of ATO-resistant CML cells (K-562) and identified UNC13B as potentially responsible for ATO resistance. UNC13B knockdown remarkably inhibited proliferation and stimulated apoptosis of K-562 cells. Western blot further revealed that UNC13B might modulate both apoptosis and mitochondrial fusion by regulating MAP3K7, CDK4, and PINK1, rendering resistance to ATO. Zhuang et al. provide a comprehensive overview of the molecular mechanisms of resistance to isocitrate dehydrogenase (IDH) inhibitors in acute myeloid leukemia (AML). A deeper understanding of the resistance mechanisms will provide rationales for novel therapeutic strategies targeting mutant IDH1/2. Several studies have demonstrated the effectiveness of homoharringtonine (HHT) for the treatment of AML. Zhang et al. profile transcriptome changes in AML cells treated with HHT and identify key metabolic pathways that may contribute to resistance to HHT in AML cells.

Collectively, the original research and review articles in this Research Topic cover a series of meaningful findings of metabolic reprogramming in hematological malignancies, providing new insights into the mechanisms underlying cellular metabolism in hematological malignancies. Moreover, several potential effective targets for cancer treatment have been proposed, and further validation is warranted.

## Author contributions

XH drafted the editorial. MKK, ZZ, and JY edited the editorial. XH and HS finalized the editorial. All authors contributed to the article and approved the submitted version.

## Funding

Financial support from the Science and Engineering Research Board Department of Science and Technology, Government of India as TARE-SERB to MKK (SERB/F/13036/2018-2019), and a grant from the Indian Council of Medical Research (ICMR), New Delhi (sanction #: 5/13/55/2020/NCD-III) are thankfully acknowledged.

## Conflict of interest

The authors declare that the research was conducted in the absence of any commercial or financial relationships that could be construed as a potential conflict of interest.

## Publisher’s note

All claims expressed in this article are solely those of the authors and do not necessarily represent those of their affiliated organizations, or those of the publisher, the editors and the reviewers. Any product that may be evaluated in this article, or claim that may be made by its manufacturer, is not guaranteed or endorsed by the publisher.

